# *Vdbgl1* Encodes a GH55 Glucan 1,3-β-Glucosidase Required for Full Virulence of *Verticillium dahliae*

**DOI:** 10.3390/ijms27156737

**Published:** 2026-07-28

**Authors:** Ruixiang Yuan, Yuanjing Li, Yongtai Li, Tiange Sun, Ao Feng, Ningbo Sun, Shuai Zhang, Qiuwei Liang, Feng Liu, Xinyu Zhang, Jie Sun, Yanjun Li

**Affiliations:** College of Agriculture, Shihezi University, Shihezi 832003, China; yuanruixiang@stu.shzu.edu.cn (R.Y.); liyuanjing@stu.shzu.edu.cn (Y.L.);

**Keywords:** *Verticillium dahliae*, cotton, glucan 1,3-β-glucosidase, cell wall-degrading enzymes, pathogenic mechanisms

## Abstract

Cotton is an economically important cash crop severely affected by Verticillium wilt caused by *Verticillium dahliae*. Secreted cell-wall-degrading enzymes act as key virulence factors of this pathogen, yet the biological function of glucan 1,3-β-glucosidase remains largely uncharacterized. The gene VDAG_02814 (designated *Vdbgl1*) was previously found to be strongly induced during host infection. Here, combined approaches including gene knockout, host-induced gene silencing (HIGS), and transcriptomic analysis were utilized to characterize the function of *Vdbgl1*. The results showed that *Vdbgl1* deletion retarded colony growth on PDA medium by 20.1–21.6%, decreased conidial yield by 20.2–46.7%, lowered spore germination rate by 30.7–32.9%, and weakened utilization of diverse carbon sources by 5.0–14.9%. The Δ*Vdbgl1* mutants also exhibited significantly increased sensitivity to cell wall-perturbing, osmotic, and membrane-damaging agents. Additionally, the Δ*Vdbgl1* mutants exhibited reduced disease index by approximately 19.1–27.7% and decreased fungal biomass by 42.7–61.8% in cotton; consistently, HIGS-mediated silencing of *Vdbgl1* reduced the disease index by 25.7% and 26.7% and decreased fungal biomass by 48.1–71.5%. Transcriptome profiling of cotton roots infected by the Δ*Vdbgl1* mutant and the wild-type strain revealed 1007 down-regulated genes enriched in carbohydrate metabolism, cell wall degradation, and energy pathways, including 27 carbohydrate-active enzyme genes and 58 genes encoding cysteine-rich secreted proteins. Collectively, these findings indicated that *Vdbgl1* coordinates carbon utilization, cell wall remodeling, and stress responses to regulate fungal development and pathogenicity, representing a promising target for cotton Verticillium wilt control.

## 1. Introduction

Cotton is an indispensable economic crop worldwide that provides major natural fiber, oil, and feed resources and occupies a critical position in China’s agricultural economy [1]. However, cotton Verticillium wilt, a destructive soil-borne vascular disease caused by *Verticillium dahliae*, severely limits cotton production [2]. This disease impairs normal cotton growth, resulting in substantial yield loss and deteriorated fiber quality. As a broad-host-range phytopathogen, *V. dahliae* can infect over 400 dicotyledonous plants and cause severe economic losses globally [3,4]. The pathogen can persist in soil and plant debris for decades in the form of microsclerotia, which are highly tolerant to adverse environments and make the disease difficult to eradicate in field conditions [5]. Under suitable conditions, soil microsclerotia germinate and produce hyphae that penetrate cotton roots. The pathogen subsequently colonizes plant vascular tissues and blocks xylem transport of water and nutrients, ultimately causing leaf yellowing, wilting, necrosis, defoliation, and even plant death [6,7]. Effective control measures are currently unavailable due to the complex pathogenic mechanisms of *V. dahliae*. Therefore, identifying pathogenicity-related genes and elucidating the underlying molecular mechanisms are essential for the prevention and control of this disease.

The plant cell wall acts as the primary physical defense barrier against pathogen invasion [8]. To overcome this obstacle, phytopathogens secrete diverse cell wall-degrading enzymes (CWDEs) during infection. These enzymes degrade core cell wall components, including cellulose, hemicellulose, and pectin, disrupting host cell wall structure and enabling pathogen invasion and colonization [9]. Previous studies have confirmed that *V. dahliae* secretes numerous extracellular virulence factors to facilitate host infection. For example, the xylanase gene *VdXyn4* degrades multiple cell wall components and modulates host immune responses at the late infection stage [10]; the pectate lyase VdPEL1 induces robust programmed cell death in *Nicotiana benthamiana* in an enzymatic activity-dependent manner, and its targeted deletion significantly compromises *V. dahliae* virulence on both tobacco and cotton [11]; the cellulases VdEG1 and VdEG3 trigger enzymatic activity-independent programmed cell death and PAMP-triggered immunity (PTI) in *N. benthamiana*, while their disruption drastically attenuates *V. dahliae* pathogenicity on cotton [12]. Collectively, these studies demonstrated that *V. dahliae* adopts a sophisticated dual infection strategy: it degrades plant cell walls via CWDEs and manipulates host immune responses through secretory effectors. The synergistic effect of these virulence factors facilitates fungal colonization and infection, underlying the complex pathogenic mechanisms of *V. dahliae* [13,14].

1,3-β-glucan is a vital plant defense polysaccharide that accumulates between the cell wall and plasma membrane upon pathogen infection and wounding, serving as a core component of the plant physical defense barrier and a major degradation target of invasive pathogenic fungi [15,16]. Glucan 1,3-β-glucosidase is a specific hydrolase that cleaves the glycosidic bonds of 1,3-β-glucan. In pathogen–host interactions, this enzyme can hydrolyze host defense-related 1,3-β-glucan, thereby disrupting callose-based defensive structures and compromising plant physical barriers to facilitate fungal invasion and colonization [17]. Concurrently, it modulates endogenous 1,3-β-glucan in fungal cell walls, thereby maintaining cell wall integrity, hyphal growth, morphogenesis, and pathogenicity [18,19]. Numerous studies have verified that glucan 1,3-β-glucosidase acts as a critical virulence factor in diverse plant-pathogenic fungi. For instance, *Pyricularia oryzae* Ebg1 triggers plant cell death, and deletion of its encoding gene drastically reduces fungal virulence to rice and barley [20]. In addition, the core effector Erc1 of *Ustilago maydis* exhibits glucan 1,3-β-glucosidase activity to inhibit β-glucan-triggered plant immunity and is indispensable for maize smut pathogenesis [21].

Previous secretome data in our lab revealed that the gene VDAG_02814 (*Vdbgl1*), which encodes a glucan 1,3-β-glucosidase, was significantly up-regulated during cotton infection by a highly virulent *V. dahliae* strain, suggesting its potential involvement in the pathogenic process [22]. To elucidate the functional characteristics and mechanism of action of *Vdbgl1*, this study systematically analyzed its roles in the growth and development, stress response, and pathogenicity of *V. dahliae* through gene knockout mutant construction, phenotypic analysis, and plant infection assays. Furthermore, this study reveals the biological function of *Vdbgl1* during host infection and provides preliminary clues for exploring the regulatory network underlying the pathogenicity of *V. dahliae*, as well as identifying a crucial candidate gene for developing eco-friendly control strategies against cotton Verticillium wilt.

## 2. Results

### 2.1. Bioinformatics Analysis of the Vdbgl1 Gene

**Figure 1:**
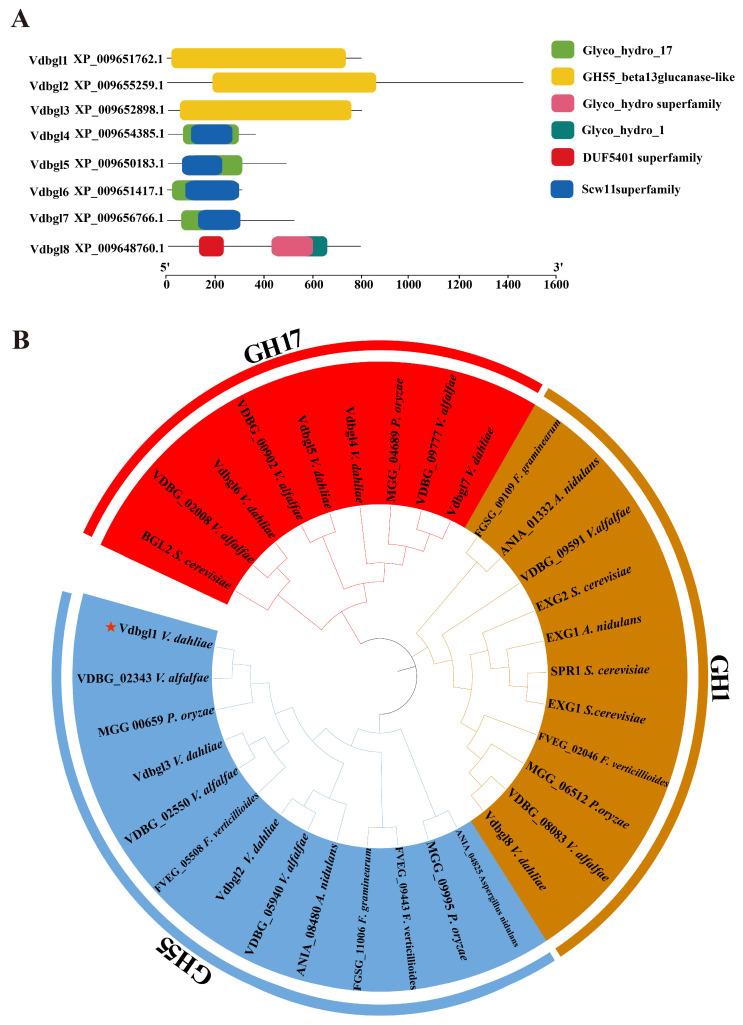
Analysis of conserved domains and phylogenetic tree of glucan 1,3-β-glucosidase. Conserved domain of 8 Vdbgl proteins in *V. dahliae*. (**A**) Conserved domain of 8 Vdbgl proteins in *V. dahliae*. Conserved domain prediction was conducted using the NCBI-CDD and SMART databases. The plot shows the conserved domain composition of each protein and their relative positions along the full-length amino acid sequences. (**B**) Phylogenetic tree of glucan 1,3-β-glucosidase genes. The tree depicts the evolutionary relationships of glucan 1,3-β-glucosidases from *V. dahliae* and other fungal species, including *Verticillium alfalfae*, *Aspergillus nidulans*, *Pyricularia oryzae*, *Fusarium graminearum*, *Saccharomyces cerevisiae*, and *Fusarium verticillioides*. The tree was constructed using the maximum likelihood method in MEGA 11.0 software, with statistical support evaluated via 1000 bootstrap replicates. Three subfamilies of glucan 1,3-β-glucosidases are indicated with three distinct colors. The *Vdbgl1* gene from *V. dahliae* characterized in this study is marked with a red star.

Based on previous secretome data in our lab, the glucan 1,3-β-glucosidase encoded by VDAG_02814 was significantly up-regulated in cotton during infection with the highly virulent *V. dahliae* strain, implying a potential role in *V. dahliae* pathogenicity. This gene was named *Vdbgl1*, which possesses a 2391 bp open reading frame (ORF). It encodes a 796-amino-acid protein with a predicted molecular weight of 85.6 kDa and a theoretical isoelectric point of 5.25. Homology analysis identified 7 additional glucan 1,3-β-glucosidase genes in the *V. dahliae* genome. In total, 8 *V. dahliae* glucan 1,3-β-glucosidases were classified into three glycoside hydrolase families via conserved domain analysis. Among them, Vdbgl1/2/3 contain a GH55_beta13glucanase-like domain; Vdbgl4/5/6/7 harbor a Glyco_hydro_17 domain; and Vdbgl8 carries a Glyco_hydro_1 domain. To clarify the evolutionary relationships of these glucan 1,3-β-glucosidases, a phylogenetic tree was constructed based on the amino acid sequences of 8 *V. dahliae* glucan 1,3-β-glucosidases and 25 homologous proteins from 6 fungal species, including *Verticillium alfalfae*, *Aspergillus nidulans*, *Pyricularia oryzae*, *Fusarium graminearum*, *Saccharomyces cerevisiae*, and *Fusarium verticillioides*. All 33 proteins were grouped into three distinct clades corresponding to the GH1, GH17, and GH55 families (Figure 1B). The GH1 family contained 11 members, including Vdbgl8, the GH17 family included 9 members, including Vdbgl4/5/6/7, and the GH55 family included 13 members, including Vdbgl1/2/3.

### 2.2. Effects of Host-Induced Silencing of Vdbgl1 on Cotton Disease Symptoms

**Figure 2:**
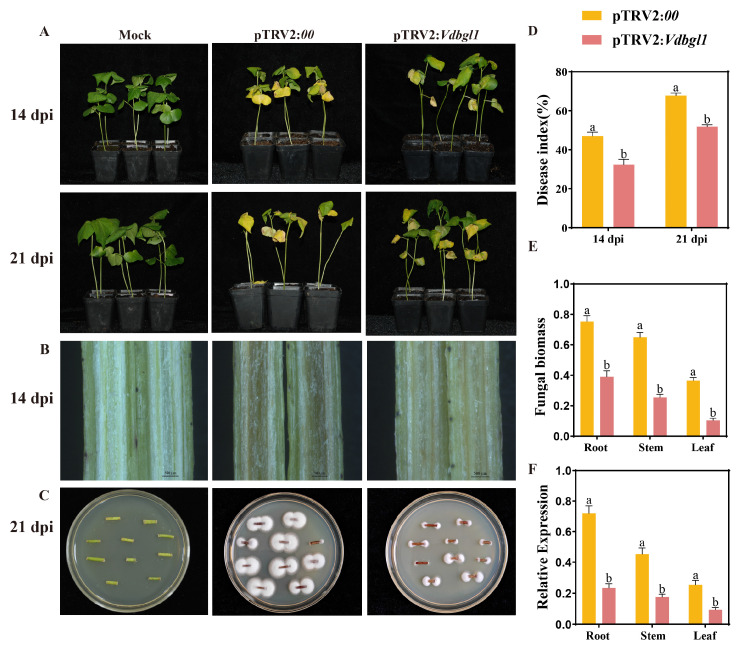
Functional analysis of *Vdbgl1* gene in the pathogenicity of *V. dahliae* using HIGS. (**A**) Disease symptoms in *Vdbgl1*-silenced (pTRV2:*Vdbgl1*) and control (pTRV2:*00*) cotton plants at 14 and 21 dpi. (**B**) Vascular browning in longitudinal stem sections of HIGS-treated plants at 14 dpi. (**C**) Fungal re-isolation from stem segments of HIGS-treated plants cultured on PDA for 7 days at 25 °C. (**D**) Disease index in *Vdbgl1*-silenced plants at 14 and 21 dpi. Disease index was calculated as: DI = [Σ(disease rating × number of plants at that rating)/(total number of plants × 4)] × 100. (**E**) Fungal biomass in different tissues of HIGS-treated plants at 21 dpi detected via qRT-PCR with *GhUBQ7* as the reference gene. (**F**) Relative expression of *Vdbgl1* in roots, stems and leaves of HIGS-treated plants analyzed via qRT-PCR using *V. dahliae β-tubulin* as the reference gene. All experiments were performed with three independent biological replicates, and data are presented as mean ± SD. Different lowercase letters on the bars indicate significant differences between treatments at the *p* < 0.05 level.

A TRV-based host-induced gene silencing (HIGS) system was employed to silence *Vdbgl1* in *V. dahliae*. The albino phenotype of cotton seedlings treated with the pTRV2:*GhCHLI* at 10 days post-treatment verified the successful establishment of the HIGS system. When cotton seedlings treated with pTRV2:*00* and pTRV2:*Vdbgl1* reached the two-leaf and one-bud stage, they were inoculated with the wild-type strain Vd991, and disease symptoms were assessed at 14 and 21 days post-inoculation (dpi). Phenotypic observations showed that cotton seedlings treated with pTRV2:*00* exhibited more severe leaf wilting and chlorosis than *Vdbgl1*-silenced plants at both detection time points (Figure 2A). Consistent with the phenotypic differences, the *Vdbgl1*-silenced seedlings displayed significantly lower disease indices (36.2 at 14 dpi and 51.2 at 21 dpi) than the control plants (48.7 at 14 dpi and 69.8 at 21 dpi), with reductions of 25.7% and 26.7%, respectively (Figure 2D). Moreover, vascular browning in the stems of *Vdbgl1*-silenced cotton was markedly alleviated compared with control plants (Figure 2B). Pathogen re-isolation assays further confirmed that *V. dahliae* accumulation was significantly reduced in *Vdbgl1*-silenced cotton relative to the control (Figure 2C). Fungal biomass quantification by qRT-PCR showed that fungal biomass in roots, stems, and leaves decreased by 48.1%, 60.9%, and 71.5%, respectively (Figure 2E). Notably, the reduction in fungal biomass was more pronounced in aboveground tissues (stems and leaves) than in roots, suggesting that *Vdbgl1* silencing may particularly impair the pathogen’s ability to colonize aerial parts of the plant. qRT-PCR analysis at 21 dpi revealed reduced *Vdbgl1* transcript levels in the roots, stems, and leaves of cotton infiltrated with pTRV2:*Vdbgl1*, confirming high-efficiency silencing of the target gene (Figure 2F). In summary, HIGS-mediated silencing of *Vdbgl1* inhibited *V. dahliae* colonization and proliferation in cotton, thereby alleviating Verticillium wilt symptoms. The percentage reductions in disease index and pathogen biomass underscore that *Vdbgl1* is essential for the full pathogenicity of *V. dahliae*.

### 2.3. Effect of Vdbgl1 Knockout on the Growth and Development of V. dahliae

**Figure 3:**
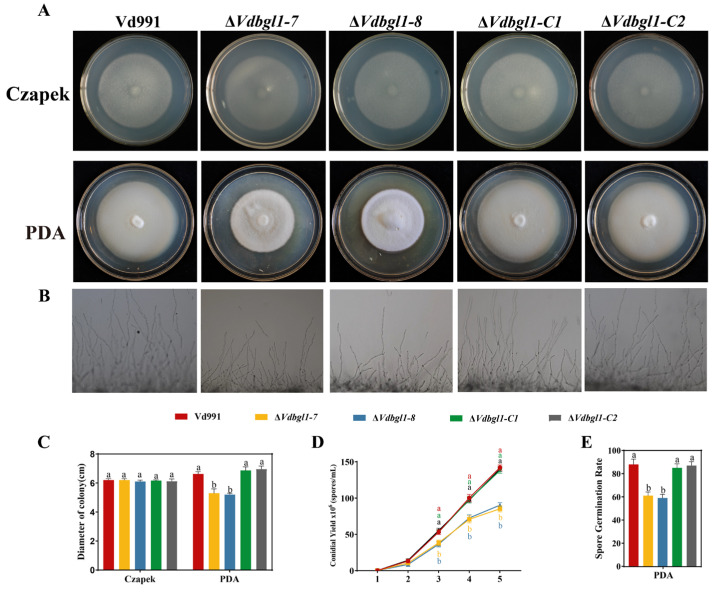
Deletion of the *Vdbgl1* gene impairs vegetative growth and development of *V. dahliae.* (**A**) Colony morphology of the wild-type strain Vd991, *Vdbgl1* knockout mutants (Δ*Vdbgl1-7*, Δ*Vdbgl1-8*), and complemented strains (Δ*Vdbgl1-C1*, Δ*Vdbgl1-C2)* incubated on Czapek and PDA media for 14 days. (**B**) Hyphal morphology at the colony margin of the tested strains after 24 h of incubation on PDA medium. (**C**) Colony diameter of the tested strains cultured on Czapek and PDA media for 14 days. (**D**) Conidial yield of the tested strains cultured in Czapek liquid medium for 5 days. (**E**) Conidial germination rate of the tested strains after 24 h of incubation on PDA medium. All experiments were performed with three independent biological replicates, and data are presented as mean ± SD. Different lowercase letters on the bars indicate significant differences between treatments at the *p* < 0.05 level.

To explore the function of *Vdbgl1* in regulating the growth and development of *V. dahliae*, *Vdbgl1* gene knockout and complemented strains were generated via *Agrobacterium tumefaciens*-mediated transformation (ATMT) based on homologous recombination, with wild-type strain Vd991 serving as the genetic background [23,24]. Two independent knockout mutants (Δ*Vdbgl1-7* and Δ*Vdbgl1-8*) and two complemented strains (Δ*Vdbgl1-C1* and Δ*Vdbgl1-C2*) were successfully constructed and verified via PCR and qRT-PCR (Figure S1). Wild-type, knockout and complemented strains were cultured on PDA and Czapek media to evaluate differences in colony morphology, vegetative growth, hyphal density, spore germination and conidial production. The results showed that Δ*Vdbgl1* mutants exhibited significantly smaller colony diameters than wild-type and complemented strains, with mycelial growth on PDA medium impaired by 20.1–21.6% (Figure 3A,C). Microscopic observation revealed that *Vdbgl1* deletion resulted in sparser peripheral hyphae compared with the control and complemented strains, suggesting compromised hyphal expansion and surface colonization capacity (Figure 3B). Furthermore, quantitative assays demonstrated that Δ*Vdbgl1* mutants produced fewer conidia, with conidial yield decreased by 20.2–46.7%, and displayed a significantly lower spore germination rate, reduced by 30.7–32.9%, relative to wild-type and complemented strains (Figure 3D,E). The consistent quantitative defects across two independent knockout mutants, together with the full restoration of growth and reproductive parameters in complemented strains, confirm that the observed phenotypes are specifically attributable to *Vdbgl1* disruption rather than off-target effects. Taken together, these results indicate that *Vdbgl1* acts as a positive regulator of growth and development in *V. dahliae*, and the pronounced reductions in vegetative growth, sporulation, and germination efficiency suggest that *Vdbgl1* is required for optimal fungal propagation and prepenetration fitness.

### 2.4. Effect of Vdbgl1 Knockout on the Pathogenicity of V. dahliae

**Figure 4:**
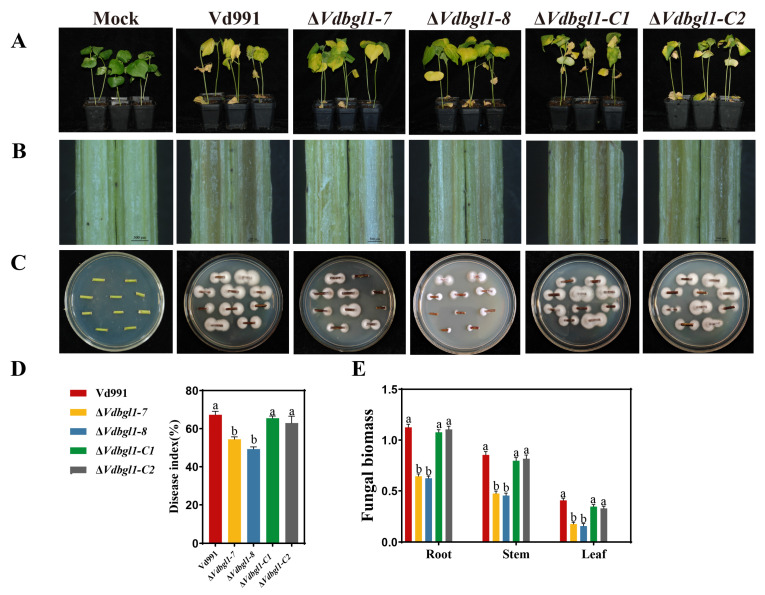
Deletion of *Vdbgl1* attenuates the virulence of *V. dahliae* on cotton. (**A**) Disease symptoms on cotton seedlings inoculated with wild-type Vd991, *Vdbgl1* knockout mutants (Δ*Vdbgl1-7*, Δ*Vdbgl1-8*), and complemented strains (Δ*Vdbgl1-C1*, Δ*Vdbgl1-C2*) at 18 dpi. (**B**) Vascular browning in longitudinal sections of cotton stems at 18 dpi. (**C**) Fungal re-isolation from infected stem segments cultured on PDA medium for 5 days. (**D**) Disease index of cotton plants inoculated with different strains. (**E**) Fungal biomass in roots, stems, and leaves of inoculated cotton plants detected via qRT-PCR with *GhUBQ7* as the reference gene. All experiments were performed with three independent biological replicates, and data are presented as mean ± SD. Different lowercase letters on the bars indicate significant differences between treatments at the *p* < 0.05 level.

To investigate the function of *Vdbgl1* in the pathogenicity of *V. dahliae*, pathogenicity assays were performed on the susceptible cotton cultivar ‘Xinluzao 7’ using wild-type strain Vd991, Δ*Vdbgl1* knockout mutants, and Δ*Vdbgl1-C* complemented strains. Phenotypic observation revealed that cotton plants infected with Δ*Vdbgl1* mutants exhibited remarkably alleviated disease symptoms, including milder leaf yellowing, wilting and defoliation, and attenuated stem vascular browning, compared with plants infected with wild-type and complemented strains (Figure 4A,B). Correspondingly, significantly lower disease indices were observed in plants infected with Δ*Vdbgl1* mutants, with disease severity reduced by approximately 19.1–27.7% relative to wild-type and complemented strains (Figure 4D). Fungal biomass quantification and re-isolation assays detected reduced fungal accumulation in plants infected with Δ*Vdbgl1* mutants, with fungal biomass decreased by 42.7–61.8% in cotton tissues (Figure 4C,E), confirming that *Vdbgl1* deletion significantly impaired the colonization and proliferation capacity of *V. dahliae* in host plants. The consistent virulence attenuation across two independent Δ*Vdbgl1* mutants, together with the full restoration of wild-type-like disease symptoms and fungal biomass in complemented strains, confirms that the reduced virulence is specifically attributable to the *Vdbgl1* disruption rather than off-target effects. Notably, the parallel reductions in disease indices and fungal biomass suggest that *Vdbgl1* is required for efficient host tissue colonization and pathogen proliferation during infection. In summary, *Vdbgl1* positively regulated the pathogenicity of *V. dahliae*, and its deletion weakened the pathogen’s ability to colonize host tissues and thereby reduced Verticillium wilt severity in cotton.

### 2.5. Effect of Vdbgl1 Knockout on Carbon Source Utilization in V. dahliae

**Figure 5:**
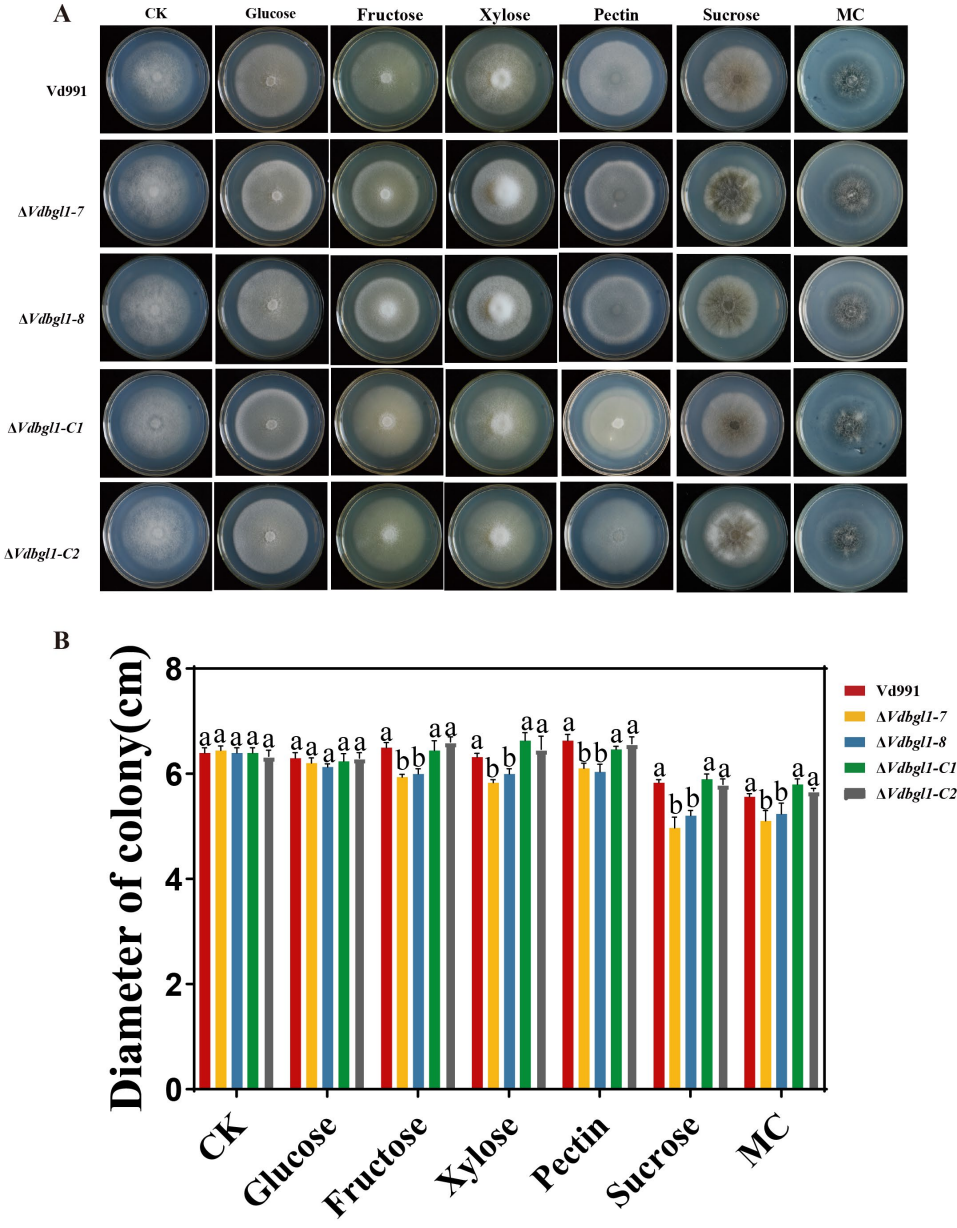
Deletion of *Vdbgl1* impairs carbon source utilization in *V. dahliae.* (**A**) Colony morphology of the wild-type strain Vd991, *Vdbgl1* knockout mutants (Δ*Vdbgl1-7*, Δ*Vdbgl1-8*), and complemented strains (Δ*Vdbgl1-C1*, Δ*Vdbgl1-C2*) after 14 days of incubation on Czapek solid medium. The medium was supplemented with glucose, fructose, xylose, pectin, sucrose, and methyl cellulose (MC) as sole carbon sources. (**B**) Colony diameters of different strains cultured under different carbon source conditions. All experiments were performed with three independent biological replicates, and data are presented as mean ± SD. Different lowercase letters on the bars indicate significant differences between treatments at the *p* < 0.05 level.

To investigate the impact of *Vdbgl1* deletion on carbon source utilization in *V. dahliae*, growth phenotypes of wild-type Vd991, Δ*Vdbgl1* knockout mutants, and Δ*Vdbgl1-C* complemented strains were compared on Czapek solid media supplemented with different sole carbon sources, including glucose, fructose, xylose, pectin, sucrose, and methyl cellulose (MC). After 14 days of cultivation, colony morphology and diameters were assessed for all strains (Figure 5A,B). No obvious differences in colony growth were observed between Δ*Vdbgl1* mutants and control strains when glucose served as the sole carbon source, indicating that *Vdbgl1* is not required for glucose metabolism. However, *Vdbgl1* deletion significantly restricted fungal growth on media containing fructose, xylose, pectin, sucrose, and methyl cellulose, as evidenced by markedly reduced colony diameters, with the utilization of these diverse carbon sources decreased by 5.0–14.9% relative to wild-type and complemented strains. The impaired growth on multiple non-glucose carbon sources suggests that *Vdbgl1* plays a broad role in fungal carbon metabolism beyond simple sugar assimilation. Notably, the compromised utilization of pectin and methyl cellulose, key structural components of plant cell walls, implies that *Vdbgl1* may facilitate nutrient acquisition from host-derived complex polysaccharides during infection. These results indicate that *Vdbgl1* participates in the utilization of multiple carbon sources and contributes to fungal nutrient adaptation and pathogenic fitness.

### 2.6. Effect of Vdbgl1 Knockout on the Stress Tolerance of V. dahliae

**Figure 6:**
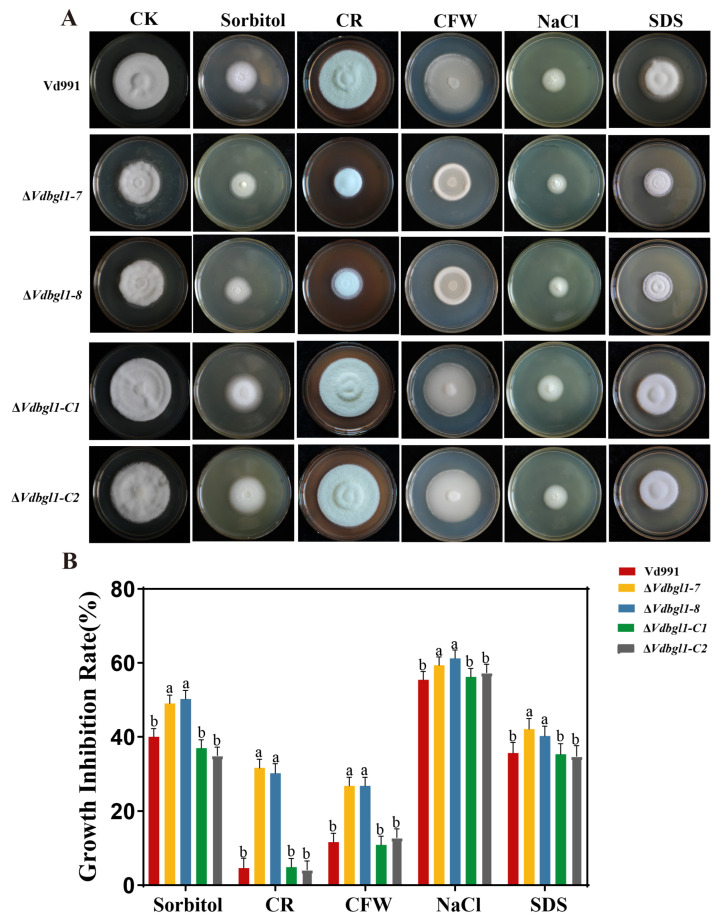
Deletion of *Vdbgl1* impairs the stress response in *V. dahliae.* (**A**) Colony phenotypes of the wild-type strain Vd991, Δ*Vdbgl1* knockout mutants (Δ*Vdbgl1-7*, Δ*Vdbgl1-8*), and complemented strains (Δ*Vdbgl1-C1*, Δ*Vdbgl1-C2*) after 14 days of incubation on PDA medium supplemented with different stress agents. (**B**) Relative growth inhibition rates of the tested strains under different stress conditions. All experiments were performed with three independent biological replicates, and data are presented as mean ± SD. Different lowercase letters on the bars indicate significant differences between treatments at the *p* < 0.05 level.

To investigate the role of *Vdbgl1* in the stress tolerance of *V. dahliae*, wild-type strain Vd991, Δ*Vdbgl1* knockout mutants, and Δ*Vdbgl1-C* complemented strains were cultured on PDA medium supplemented with distinct stress-inducing agents. Sorbitol and NaCl were applied to create osmotic stress, Congo red and calcofluor white (CFW) served as cell wall-disturbing agents, and SDS was used to trigger membrane damage stress. After 14 days of incubation, the relative growth inhibition rates of fungal strains under each treatment were quantified (Figure 6A,B). The results revealed that Δ*Vdbgl1* mutants exhibited significantly higher growth inhibition rates than wild-type and complemented strains across all tested stress conditions. Together, these findings demonstrated that *Vdbgl1* is indispensable for the stress adaptation of *V. dahliae*.

### 2.7. Prediction of the Vdbgl1 Signal Peptide and Identification of Its Secretion Function

**Figure 7:**
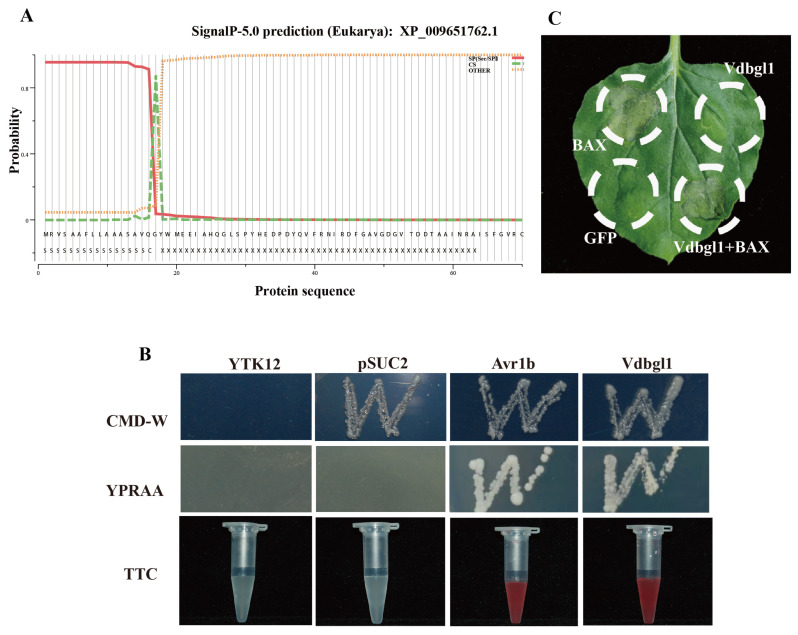
Vdbgl1 is a Secreted Protein with a Lack of Cell Death-Inducing Activity. (**A**) The predicted signal peptide site of the Vdbgl1 protein. (**B**) Functional validation of the Vdbgl1 signal peptide via the yeast signal sequence trap system. Yeast strain YTK12 was transformed with empty pSUC2 vector, pSUC2-Avr1b^SP^ (positive control), or pSUC2-*Vdbgl1*^SP^, with untransformed YTK12 as the blank control. The growth status of transformants on CMD-W and YPRAA media, and TTC staining results for secretory activity validation are shown. (**C**) Transient expression assay showing that Vdbgl1 does not induce or suppress cell death in *N. benthamiana* leaves. BAX was used as a positive control for cell death induction, and GFP as a negative control. All experiments were independently repeated at least three times.

SignalP 5.0 online prediction identified a typical N-terminal signal peptide spanning amino acid residues 1–18 of the Vdbgl1 protein (Figure 7A). A yeast signal trap system was further applied to verify the secretory function of this signal peptide [25]. The yeast strain YTK12 carrying pSUC2-*Vdbgl1*^SP^ grew normally on CMD-W medium, confirming successful transformation. The transformant also grew on YPRAA medium, indicating that the Vdbgl1 signal peptide successfully mediated the secretion of the fused invertase, which enabled the yeast to utilize raffinose for growth. In addition, the positive TTC (2,3,5-triphenyltetrazolium chloride) reaction with red insoluble triphenylformazan formation further confirmed the secretory activity of the Vdbgl1 signal peptide (Figure 7B). To explore the potential role of *Vdbgl1* in regulating plant immunity, an Agrobacterium-mediated transient expression assay was performed in *N. benthamiana* leaves. Individual expression of *Vdbgl1* did not trigger plant cell death, and co-expression of *Vdbgl1* failed to suppress BAX-induced programmed cell death. These results suggested that *Vdbgl1* is not involved in regulating plant immune responses (Figure 7C).

### 2.8. Transcriptomic Analysis of the Wild-Type and Vdbgl1 Knockout Mutants During Infection

**Figure 8:**
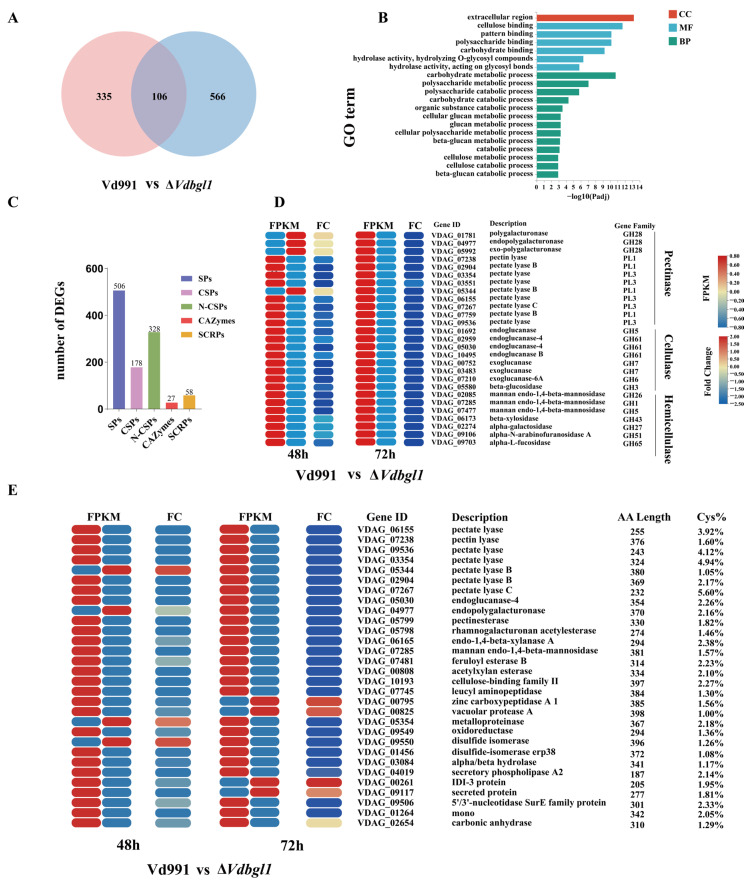
Identification of DEGs potentially associated with the attenuated pathogenicity of the Δ*Vdbgl1* mutant. (**A**) Venn diagram of down-regulated DEGs in the Δ*Vdbgl1* vs. wild-type comparison at two time points (48 and 72 hpi) after infection. (**B**) Gene Ontology (GO) enrichment analysis for the down-regulated DEGs. (**C**) Classification of genes encoding SPs among the down-regulated DEGs. (**D**) Heatmap showing the expression levels of a subset of significantly down-regulated CAZyme genes. (**E**) Heatmap showing the expression levels of a subset of significantly down-regulated SCRP genes. Transcriptome sequencing was performed with three independent biological replicates. DEGs were filtered with fold change ≥ 1.5 and adjusted *p*-value(padj) < 0.05. GO enrichment analysis was conducted using the hypergeometric test.

To investigate the role of *Vdbgl1* in the pathogenicity of *V. dahliae*, transcriptome sequencing was performed on cotton roots infected with the wild-type strain Vd991 and the Δ*Vdbgl1* mutant strain. Clean sequencing reads were mapped to the reference genome of *V. dahliae*. Eight genes were randomly selected for qRT-PCR validation, which confirmed the reliability of the RNA-seq results (Figure S2). Transcriptomic comparison identified a total of 1007 down-regulated differentially expressed genes (DEGs) in the cotton roots infected with the Δ*Vdbgl1* mutant at 48 and 72 h post-inoculation (hpi), including 335 DEGs at 48 hpi, 566 DEGs at 72 hpi, and 106 common DEGs at both time points (Figure 8A). Gene Ontology (GO) enrichment analysis was conducted for these 1007 down-regulated DEGs. In the cellular component category, DEGs were significantly enriched in the extracellular region. For molecular function, the enriched terms primarily included cellulose binding, pattern binding, and polysaccharide binding. In the biological process category, carbohydrate metabolic process, polysaccharide metabolic and catabolic processes were the most highly enriched pathways (Figure 8B).

Among the 1007 down-regulated DEGs, 506 were predicted to encode secreted proteins (SPs), consisting of 178 classical secreted proteins (CSPs) and 328 non-classical secreted proteins (N-CSPs) (Figure 8C). The 178 CSPs contained 27 carbohydrate-active enzymes (CAZymes) and 58 small cysteine-rich proteins (SCRPs, <400 amino acids and containing ≥4 cysteine residues). The 27 CAZymes were further classified into 12 pectinases, 8 cellulases, and 7 hemicellulases (Figure 8D). The 58 SCRPs mainly comprised 3 metalloproteases, 3 oxidoreductases, 2 disulfide isomerases, and 2 hydrolases; notably, 16 of these were identified as plant cell wall-degrading enzymes, including 10 pectinases, 2 cellulases, and 4 hemicellulases (Figure 8E).

## 3. Discussion

Glycoside hydrolases (GHs) are the largest family of CAZymes and are classified into over 100 subfamilies (GH1–GH171) in the CAZy database based on sequence characteristics, protein structure, and catalytic mechanisms [26,27]. GHs primarily hydrolyze glycosidic bonds and participate in polysaccharide degradation, carbon metabolism, cell wall remodeling, and pathogen–host interactions [28,29]. Among them, the GH55 family specifically acts on 1,3-β-glucan substrates, including exo-1,3-β-glucanases (EC 3.2.1.58) and endo-1,3-β-glucanases (EC 3.2.1.39) that synergistically mediate glucan chain cleavage and degradation [18]. GH55 proteins are highly conserved in filamentous fungi and govern cell wall remodeling, spore development, hyphal morphogenesis. For example, the GH55 member in *Aspergillus fumigatus* is critical for conidial wall assembly, and its deletion causes defective spore development, delayed germination, and reduced virulence [30]. In a previous study, the GH55 family 1,3-β-glucosidase *Vdbgl1*, containing an N-terminal signal peptide, was identified from the secretome data of *V. dahliae*. In this study, gene deletion mutants and complemented strains were generated, confirming that *Vdbgl1* is essential for fungal growth, development, carbon source utilization, stress tolerance, and pathogenicity.

Accumulating evidence has confirmed that GH family proteins contribute to carbon source utilization in phytopathogenic fungi [31,32,33]. However, the role of 1,3-β-glucosidase genes in carbon metabolic adaptation remains largely uncharacterized. In this study, *Vdbgl1* deletion significantly impaired fungal growth when fructose, xylose, pectin, sucrose, or methyl cellulose served as the sole carbon source, demonstrating that *Vdbgl1* is essential for the utilization of diverse carbon substrates in *V. dahliae* (Figure 5). Transcriptomic analysis further revealed that loss of *Vdbgl1* markedly down-regulated numerous genes encoding pectinases, cellulases, and hemicellulases (Figure 8). These cell wall-degrading CAZymes are indispensable for fungal degradation of plant polysaccharides and carbon nutrient acquisition. The suppression of these enzyme genes disrupts the core carbohydrate metabolic network, which explains the compromised carbon utilization capacity of Δ*Vdbgl1* mutants. 

Glucan 1,3-β-glucosidase is a core enzyme for fungal cell wall remodeling that modulates dynamic 1,3-β-glucan turnover to maintain cell wall stability and metabolic homeostasis [34,35]. Intact cell wall structure is essential for pathogenic fungi to resist environmental stresses and host immune attacks [36]. Previous studies in phytopathogenic fungi, including *P. oryzae* and *A. fumigatus*, have demonstrated that the deletion of glucan 1,3-β-glucosidase family genes leads to abnormal cell wall structure, increased stress sensitivity, and significantly reduced virulence [20,30]. Consistent with these findings, our stress assays showed that Δ*Vdbgl1* mutants exhibited significantly higher growth inhibition than wild-type and complemented strains under multiple stress conditions (Figure 6). The Δ*Vdbgl1* mutants displayed severe growth defects under treatment with the cell wall-perturbing agents CR and CFW, indicating that *Vdbgl1* deletion compromises cell wall structural integrity in *V. dahliae*. In addition, Δ*Vdbgl1* mutants were more sensitive to sorbitol- and NaCl-induced osmotic stress, suggesting that the *Vdbgl1* deletion may indirectly weaken the adaptability of *V. dahliae* to various environmental stresses by impairing cell wall integrity.

Notably, not all secreted glycoside hydrolases function as PAMPs. For instance, among the six GH12 proteins identified in *V. dahliae*, only VdEG1 and VdEG3 triggered plant immune responses, whereas the remaining members, including VdEG2 and VdEG4, failed to induce cell death or ROS accumulation despite being successfully expressed [12]. Similarly, the β-1,3-glucanase CgEC124 from *Colletotrichum graminicola* was shown to lack PAMP activity and could not elicit ROS burst in planta [17]. Consistent with these observations, Vdbgl1, although possessing a functional N-terminal signal peptide and being secreted, failed to induce plant cell death or suppress BAX-triggered programmed cell death in transient expression assays (Figure 7). This phenotype is likely attributed to the primary function of Vdbgl1 as a cell wall-degrading glucosidase, rather than acting as a canonical PAMP or a cytoplasmic effector that directly engages with plant immune recognition systems. Interestingly, although Vdbgl1 does not appear to function as a direct immune modulator, its deletion substantially down-regulated the expression of numerous effector-encoding genes, implying that Vdbgl1 may indirectly influence effector production by sustaining fungal cell wall integrity and metabolic fitness—prerequisites for the proper expression and secretion of virulence factors during infection.

Effector proteins are core virulence factors that mediate pathogen–host interactions, enabling pathogenic fungi to break through plant immune barriers, perturb host defense responses, and achieve successful colonization and proliferation in host tissues [37,38]. In *V. dahliae*, effectors facilitate infection by suppressing plant immune signaling, eliminating reactive oxygen species bursts, and inhibiting host cell wall defense [39,40]. In this study, comparative transcriptomic analysis of infected cotton roots revealed extensive down-regulation of effector-encoding genes (506 secreted proteins) in Δ*Vdbgl1* mutants (Figure 8). These repressed genes included well-characterized virulence effectors, such as pectate lyases, cellulases, and hemicellulases that mediate plant cell wall degradation [41,42], as well as uncharacterized SCRPs, whose functions in *V. dahliae* pathogenicity remain to be explored [38,43]. The widespread down-regulation of effector genes indicated that *Vdbgl1* deficiency may impair the fungal capacity to suppress host immunity and disrupt defense pathways during infection, leading to substantially reduced colonization, expansion, and accumulation in the host. Therefore, these down-regulated effectors also provide a molecular basis for the significantly attenuated virulence of the Δ*Vdbgl1* mutant.

In summary, this study systematically characterized the biological functions of the GH55-family 1,3-β-glucosidase gene *Vdbgl1* in *V. dahliae*. *Vdbgl1* positively regulated fungal growth, development, carbon metabolism, and stress adaptation to support full virulence. Deletion of *Vdbgl1* impaired hyphal growth, conidiation, and spore germination, disrupted cell wall integrity and stress tolerance, and compromised the utilization of multiple carbon sources required for infection. Transcriptomic evidence further revealed that loss of *Vdbgl1* substantially repressed the expression of numerous CAZymes and secretory effector genes, including cell wall-degrading enzymes and uncharacterized SCRPs, which were essential for plant cell wall degradation and host immune suppression. Collectively, these multi-layered defects severely restricted fungal colonization and proliferation in cotton and reduced in planta fungal biomass, ultimately leading to markedly attenuated virulence of Δ*Vdbgl1* mutants. This study revealed the biological function of *Vdbgl1* in the pathogenicity of *V. dahliae* and identified a promising candidate target for developing eco-friendly control strategies against cotton Verticillium wilt.

## 4. Materials and Methods

### 4.1. Plant Materials and Fungal Strains

The *V. dahliae* strain Vd991, *Agrobacterium tumefaciens* strain GV3101, *Gossypium hirsutum* ‘Xinluzao 7’, a susceptible cultivar to *V. dahliae*, and *N. benthamiana* used in this study were all preserved in the Key Laboratory of Oasis Eco-agriculture, College of Agriculture, Shihezi University. The vectors for virus-induced gene silencing (VIGS), pTRV1, pTRV2, and pTRV2:*GhCHLI*, were kindly provided by Professor Longfu Zhu of Huazhong Agricultural University. The plant expression vector pGR107 was kindly provided by Professor Hongjie Feng of the Institute of Cotton Research, Chinese Academy of Agricultural Sciences.

Vd991 is a highly virulent defoliating strain. The strain was inoculated into liquid Czapek medium and cultured at 25 °C with shaking at 150 rpm for 5–7 days. After cultivation, the mycelia were filtered through eight layers of sterile gauze, and conidia were collected. The conidial suspension was counted using a hemocytometer and adjusted to a concentration of 1.0 × 10^7^ conidia/mL with sterile water.

‘Xinluzao 7’ plants were grown in a growth chamber under conditions of 25 °C with a 14 h/10 h (light/dark) photoperiod. *N. benthamiana* plants were grown in a growth chamber at 25 °C with a 16 h/8 h (light/dark) photoperiod and 60–70% relative humidity.

### 4.2. DNA and RNA Extraction, and cDNA Synthesis

Genomic DNA from *V. dahliae* was extracted using the Fungal DNA Kit (Omega Bio-tek, Norcross, GA, USA). Total RNA from *V. dahliae* was extracted using the Fungal RNA Kit (Omega Bio-tek, Norcross, GA, USA), and total RNA from cotton tissues was extracted using the EASYspin Plus Plant RNA Extraction Kit (Aidlab Biotechnologies Co., Ltd., Beijing, China). All steps were performed strictly following the manufacturer’s instructions. After removing genomic DNA contamination from RNA samples, reverse transcription was performed using the One-Step gDNA Removal and cDNA Synthesis SuperMix (TransGen Biotech, Beijing, China). The synthesized cDNA was stored at −20 °C for further use.

### 4.3. Bioinformatics Analysis of the Glucan 1,3-β-Glucosidase Gene of V. dahliae

The genomic sequence (ASM15067v2), gene annotation file (gff3), and protein sequence file of *V. dahliae* were downloaded from the National Center for Biotechnology Information (NCBI, https://www.ncbi.nlm.nih.gov, accessed on 20 October 2025). Using the glucan 1,3-β-glucosidase of *V. dahliae* as a query, homologous protein sequences were retrieved using NCBI BLAST (https://blast.ncbi.nlm.nih.gov, accessed on 20 October 2025) from *V. dahliae*, *V. alfalfae*, *A. nidulans*, *P. oryzae*, *F. graminearum*, *S. cerevisiae*, and *F. verticillioides*. Multiple sequence alignment of the glucan 1,3-β-glucosidase protein sequences from *V. dahliae* and other fungi was performed using the Clustal W program in MEGA 11.0, and a phylogenetic tree was subsequently constructed using the maximum likelihood method with 1000 bootstrap replicates for statistical assessment. Conserved domain analysis was carried out using the NCBI Conserved Domain Database (CDD) batch CD-search tool, and the resulting hitdata.txt file was subjected to protein conserved domain visualization with TBtools_V1.122.

### 4.4. Host-Induced Gene Silencing of Vdbgl1

The CDS sequence of *Vdbgl1* was retrieved from the NCBI database. Specific primers HIGS-*Vdbgl1*-F/R (Table S1) were designed using the Primer3 Plus online tool (http://primer3plus.com, accessed on 20 October 2025) to amplify the silencing fragment. The target fragment was cloned into the empty vector pTRV2:*00* to generate pTRV2:*Vdbgl1*, which was then transformed into *A*. *tumefaciens* strain GV3101 by electroporation. An equal mixture (1:1, OD600nm = 0.8) of *A. tumefaciens* cultures harboring pTRV1 and pTRV2:*Vdbgl1* was infiltrated into cotton seedlings at the two-cotyledon stage as described [44]. Two weeks after infiltration, the pTRV2:*GhCHLI* plants were examined for the appearance of a photobleaching phenotype. When the cotton seedlings reached the two-true-leaf stage, they were inoculated with a conidial suspension of *V. dahliae* (1 × 10^7^ conidia/mL) via a root-dip method.

### 4.5. Gene Knockout and Complementation of Vdbgl1

To construct the *Vdbgl1* knockout vector, the upstream flanking sequence (1015 bp) and downstream flanking sequence (983 bp) of *Vdbgl1* were amplified from Vd991 genomic DNA using primer pairs *Vdbgl1*-UP-F/R and *Vdbgl1*-Down-F/R, respectively (primer sequences are listed in Table S1). After purification, the amplified fragments were cloned into the *Pac*I-linearized (Vazyme Biotech Co., Ltd., Nanjing, China) pGKO-HPT vector using the ClonExpress II One Step Cloning Kit C115 (Vazyme Biotech Co., Ltd., Nanjing, China) to generate the *Vdbgl1* knockout vector. The validated recombinant vector was then introduced into the wild-type strain Vd991 via Agrobacterium tumefaciens-mediated transformation (ATMT) as described previously [23,24]. Transformants were selected on PDA medium containing hygromycin B (Coolaber, Beijing, China) and verified via PCR with primer pairs Hyg-F/R and *Vdbgl1*-F/R. The resulting *Vdbgl1* deletion mutant was designated Δ*Vdbgl1*.

To construct the complementation vector, a DNA fragment (3866 bp) encompassing the *Vdbgl1* promoter and full-length coding sequence was amplified from Vd991 genomic DNA using primer pairs Promoter-F/R and *Vdbgl1*-F/R. The purified fragment was cloned into the pCAMBIA1302 vector using the ClonExpress II One Step Cloning Kit C115 to generate the *Vdbgl1* complementation vector. The validated complementation vector was transformed into the Δ*Vdbgl1* mutant by ATMT. Transformants were selected on PDA medium containing G418 (Coolaber, Beijing, China) and verified via PCR with primers *Vdbgl1*-F/R. The complemented strain was designated Δ*Vdbgl1-C*.

### 4.6. Effect of Vdbgl1 on Fungal Growth and Development

The wild-type strain Vd991, the Δ*Vdbgl1* deletion mutant, and the complemented strain Δ*Vdbgl1-C* were inoculated into Czapek medium and cultured at 25 °C for 5 d. Conidia were collected and adjusted to 1 × 10^7^ conidia/mL with sterile water. A 10 μL aliquot of the conidial suspension was inoculated onto PDA plates and incubated upside down at 25 °C for 14 d. Colony diameter was measured using the cross method and colony morphology was recorded. A 10 μL droplet of the conidial suspension (1 × 10^7^ conidia/mL) was placed on a sterile glass slide, incubated in a moist chamber at 25 °C for 24 h, and then examined under a microscope to determine the germination rate. To measure conidial production, 200 μL of the conidial suspension (1 × 10^7^ conidia/mL) was added to 50 mL of Czapek medium and incubated at 25 °C in the dark with shaking at 220 rpm. Samples were collected at 1, 2, 3, 4, and 5 d for conidial counting. All experiments were repeated three times.

### 4.7. Carbon Source Utilization Analysis

To analyze the carbon source utilization of the wild-type strain Vd991, the Δ*Vdbgl1* deletion mutant, and the complemented strain Δ*Vdbgl1-C*, glucose (10 g/L), fructose (10 g/L), xylose (10 g/L), pectin (10 g/L), sucrose (10 g/L), and methylcellulose (10 g/L) were individually added to sucrose-deficient Czapek–Dox agar medium [45]. Czapek–Dox agar medium without any carbon source served as the control. All plates were incubated at 25 °C in the dark for 14 d, after which colony diameter was measured. All experiments were repeated three times.

### 4.8. Stress Tolerance Assays

To evaluate the sensitivity of the wild-type strain Vd991, the Δ*Vdbgl1* deletion mutant, and the complemented strain Δ*Vdbgl1-C* to cell wall perturbing agents and osmotic stress, each strain was cultured on PDA plates supplemented with 1 M sorbitol, 0.02% Congo red (CR), 30 μg/mL calcofluor white (CFW), 1 M NaCl, or 0.002% sodium dodecyl sulfate (SDS). PDA plates without any supplement served as the control. All plates were incubated at 25 °C in the dark for 14 d, after which colony diameters were measured and the relative growth inhibition rate was calculated using the formula: (colony diameter of control − colony diameter of treatment)/colony diameter of control × 100% [46]. Each experiment was repeated three times.

### 4.9. Pathogenicity Assay of the Vdbgl1 Deletion Mutant

To compare pathogenicity among strains, two-true-leaf cotton seedlings were inoculated via the root-dip method with conidial suspensions (1 × 10^7^ conidia/mL) of wild-type Vd991, the Δ*Vdbgl1* mutant and the complemented strain Δ*Vdbgl1-C*. Disease severity was evaluated at 18 d post-inoculation using a 0–4 disease rating scale. The disease index was calculated using the following formula: disease index = [Σ (number of leaves per disease grade × disease grade value)/(total number of plants × 4)] × 100 [47].

Eighteen days after inoculation, diseased plants were selected, and stem segments approximately 3 cm above the root were excised. The segments were longitudinally sectioned along the vascular bundles, observed under a stereomicroscope (SteREO Discovery.V8; Carl Zeiss Microscopy GmbH, Jena, Germany) for the degree of vascular browning, and photographed. Simultaneously, young stems of diseased plants were surface-sterilized with 5% NaClO (Wodeer Biotechnology Co., Ltd., Shihezi, China), and 0.2 cm was trimmed from both ends. The remaining stems were cut into segments of approximately 1 cm, placed evenly onto PDA plates, and incubated at 25 °C for 3–7 d. The colony morphology was then observed and recorded.

To quantify the fungal biomass in plants, root, stem, and leaf tissues were collected from three cotton seedlings at 18 d post-inoculation. Total RNA was extracted and reverse-transcribed into cDNA. qRT-PCR was performed using the cotton *GhUBQ7* gene as the internal reference and the fungi-specific primer pair *ITS1*-F/*ST*-*Ve1*-R. The relative biomass of the pathogen in different host tissues was subsequently calculated.

### 4.10. Functional Validation of the Vdbgl1 Signal Peptide

The yeast signal sequence trap system was used to validate the secretory function of the predicted signal peptide of the *Vdbgl1* gene. Using cDNA from Vd991 as the template, the signal peptide-encoding sequence of *Vdbgl1* was amplified via PCR with the primers pSUC2-*Vdbgl1*^SP^-F/R (Table S1). The amplicon was integrated into the pSUC2 vector via restriction enzyme digestion and ligation to generate the recombinant plasmid pSUC2-*Vdbgl1*^SP^, which was then transformed into the yeast strain YTK12. The empty vector pSUC2 and pSUC2-Avr1b^SP^ served as negative and positive controls, respectively. Positive clones were selected on tryptophan-deficient CMD-W medium and subsequently cultured on YPRAA medium containing 2% raffinose (Coolaber, Beijing, China). The signal peptide-mediated invertase activity was assessed using the 2,3,5-triphenyltetrazolium chloride (TTC, Coolaber, Beijing, China) colorimetric assay. Invertase hydrolyzes sucrose into glucose and fructose, and the colorless TTC is reduced to red formazan crystals under reducing conditions [48]; the color intensity is positively correlated with the enzymatic activity. The secretion function of the signal peptide was determined by observing the TTC color change.

### 4.11. Agrobacterium-Mediated Transient Transformation in N. benthamiana

The full-length CDS of *Vdbgl1*, including the signal peptide-encoding sequence, was amplified from Vd991 cDNA using primers pGR107-*Vdbgl1*-F/R (primer sequences are listed in Table S1). The amplicon was digested and ligated into the pGR107 expression vector to generate the recombinant plasmid pGR107:*Vdbgl1*. The resulting construct was introduced into *A*. *tumefaciens* GV3101 by the freeze-thaw method. For transient expression assays in *N. benthamiana*, *A. tumefaciens* GV3101 cells harboring the pGR107 constructs were resuspended in MES buffer (10 mM MgCl_2_, 10 mM MES, 10 μM acetosyringone) and incubated in the dark for 2–3 h following previously described methods [48]. The bacterial suspensions were adjusted to an OD_600_nm of 0.8–1.0 and infiltrated into leaves of 5-week-old *N. benthamiana* plants. Leaves were sampled 5–7 d post-infiltration, and the cell death phenotype was observed and recorded. *A. tumefaciens* GV3101 carrying pGR107:GFP was used as a negative control, and GV3101 carrying pGR107:BAX served as a positive control. To investigate the potential cell death-suppressing function of *Vdbgl1*, *A. tumefaciens* strains harboring pGR107:*Vdbgl1* and pGR107:BAX were mixed at a 1:1 ratio and co-infiltrated into *N. benthamiana* leaves.

### 4.12. RNA Sequencing

At the two-true-leaf stage, cotton seedlings were wounded at the roots and inoculated by immersing roots in conidial suspensions (1 × 10^7^ conidia/mL) of wild-type Vd991 or Δ*Vdbgl1* mutant; mock controls were treated with sterile water. Root tips and root hair zones (2–3 cm) were collected at 48 and 72 hpi, rinsed with sterile water, immediately snap-frozen in liquid nitrogen, and stored at −80 °C. Three biological replicates were prepared for each treatment and subjected to RNA-seq analysis. RNA-seq experiments and data analysis were performed by Nanjing Personalbio Gene Technology Co., Ltd. (Nanjing, China). Three independent biological replicates were established for each experimental condition. Total RNA was extracted, followed by quality assessment and integrity evaluation. Qualified RNA samples were used for cDNA library construction and then subjected to paired-end 150 bp (PE150) sequencing on an Illumina platform. Raw reads were quality-controlled using Fastp_V0.22.0 to remove adapter contamination, reads containing ambiguous bases (poly-N), and low-quality reads, yielding high-quality clean reads. As previously reported [49,50], clean reads were aligned to the *V. dahliae* VdLs.17 reference genome using HISAT2_V2.1.0, followed by transcript assembly and abundance estimation with StringTie_V2.2.1. Gene expression levels were normalized as fragments per kilobase of transcript per million mapped reads (FPKM).

### 4.13. Differentially Expressed Genes (DEGs) and Gene Annotation Analysis

Based on raw read counts, differentially expressed genes (DEGs) between groups were identified using DESeq2_V1.38.3 with the thresholds of |log2 fold change| > 0.585 (corresponding to a >1.5-fold change) and an adjusted *p*-value (Padj) < 0.05. GO (Gene Ontology) enrichment analysis of DEGs was performed using clusterProfiler_V4.6.0, with Padj < 0.05 considered statistically significant. In addition, secreted proteins were predicted based on SignalP 5.0 (https://services.healthtech.dtu.dk/services/SignalP-5.0/, accessed on 25 March 2026), TMHMM 2.0 (https://services.healthtech.dtu.dk/services/TMHMM-2.0/, accessed on 25 March 2026), and SecretomeP 2.0 (https://services.healthtech.dtu.dk/services/SecretomeP-2.0/, accessed on 25 March 2026) [51,52,53]. Annotation of putative CAZymes was performed using a Hidden Markov Model (HMM) routine based on the carbohydrate-active enzymes database (https://www.cazy.org/, accessed on 26 March 2026) [54].

### 4.14. Statistical Analysis

All experiments were performed in triplicate as three independent biological replicates. All data are presented as mean ± standard deviation (Mean ± SD). Data were analyzed using IBM SPSS Statistics 26.0. One-way analysis of variance (ANOVA) was conducted, and post hoc multiple comparisons were performed using Duncan’s multiple range test. Differences between treatments were considered statistically significant at the *p* < 0.05 level, and were indicated by different lowercase letters on the bars.

## 5. Conclusions

This study systematically characterized the biological function of the GH55-family glucan 1,3-β-glucosidase gene *Vdbgl1* in *V. dahliae*. *Vdbgl1* acted as a positive regulator of fungal pathogenicity, and its deletion caused comprehensive phenotypic defects, including impaired growth and development, disrupted cell wall integrity, reduced stress tolerance, and defective carbon source utilization. These abnormalities ultimately suppress the colonization and proliferation of *V. dahliae* in cotton tissues and lead to significantly attenuated fungal virulence. Transcriptomic analysis further revealed that the loss of *Vdbgl1* markedly down-regulates the expression of CAZymes and secretory effector genes associated with plant cell wall degradation and host immune suppression, which provides a molecular explanation for the weakened pathogenicity of Δ*Vdbgl1* mutants. These findings provide novel insights into the molecular basis of *V. dahliae* pathogenicity and identify a crucial candidate target for developing eco-friendly control strategies against cotton Verticillium wilt. Further studies are warranted to elucidate the molecular signaling pathways linking *Vdbgl1*-mediated cell wall remodeling to the transcriptional regulation of virulence-associated effectors and CAZymes, and to explore the utility of *Vdbgl1* as a target for disease control.

## Data Availability

The datasets presented in this study are available in online public repositories. The repository link and accession number are as follows: NCBI GEO database: https://www.ncbi.nlm.nih.gov/geo/ (accessed on 11 June 2026) accession number PRJNA1478237.

## Supplementary Materials

The following supporting information can be downloaded at: https://www.mdpi.com/article/10.3390/ijms27156737/s1.
