# Supplementary material for: Vdbgl1 Encodes a GH55 Glucan 1,3-β-Glucosidase Required for Full Virulence of Verticillium dahliae
Source: Int J Mol Sci. 2026 Jul 28;27(15):6737. doi: 10.3390/ijms27156737 (PMC13466425; doi:10.3390/ijms27156737)
Supplement: Supplementary file 1 [file ijms-27-06737-s001.zip › ijms-4411349-supplementary.pdf]

Table S1. Primers used in the study

Gene name	Primer name	Primer sequence (5′-3′)	Destination
Vdbgl1 (VDAG_02814)	HIGS-*Vdbgl1*-F	CTCGGTACCGGATCCCTCAAGATCGAGGCGTCGCG	PCR
	HIGS-*Vdbgl1*-R	CTCGGTACCGGATCCCTCGTCGTGTCTGTAAACGCCAA	PCR
	pGR107-*Vdbgl1*-F	GGTCAGCACCAGCTAGCATCGATATGCGTGTCTCGGCCGCT	PCR
	pGR107-*Vdbgl1*-R	TCGCCCTTGCTCACCATCCCGGGCTCGTCGTGTCTGTAAACGCCAA	PCR
	pSUC2-*Vdbgl1*^{SP}-F	GGAATTTTAATTAAGAATTCATGCGTGTCTCGGCCGCT	PCR
	pSUC2-*Vdbgl1*^{SP}-R	ACTATAGGGAGAACCTCGAGCTCGTCGTGTCTGTAAACGCCAA	PCR
	qRT-PCR-*Vdbgl1*-F	GCCCTCAAGATCGAGGCGT	qRT-PCR
	qRT-PCR-*Vdbgl1*-R	GAGGTTGTAGACCTCGACGCC	qRT-PCR
	Vdbgl1-Up-F	GAAGCTTGCTGAGGTCTTAATTAAGAGGTGAGGCTCAACCGTGGTC	PCR
	Vdbgl1-Up-R	GACTAGTGCTGAGGCATTAATTAACGTGGGCTTCAATGGCTCTGAC	PCR
	Vdbgl1-Down-F	TGGGCCCGCTGAGGACTTAATTAAGACTTTTCTTTTCTCCGGTTTTCT	PCR
	Vdbgl1-Down-R	CGAGCTCGCTGAGGGTTTAATTAACTCGTCGACATTGGCAAC	PCR
	Promotor-F	ACGGCCAGTGCCAAGCTTCTATTTAGCATCTGACATCGCTGCCT	PCR
	Promotor-R	ATTCACTAGTCAGGATCCACAATTCGTGGGCTTCAATGGCTCTG	PCR
Tubulin	*Tubulin*-F	TCCACCTTCGTCGGTAACTC	qRT-PCR
	Tubulin-R	GCCTCCTCCTCGTACTCCTC	qRT-PCR
VDAG_06155	qRT-PCR-06155-F	AAGGGTGTCTTCGACGGTGG	qRT-PCR
	qRT-PCR-06155-R	CTTGCAGTGGACACCCTCGA	qRT-PCR
VDAG_00752	qRT-PCR-00752-F	GTGCCATGGGATCTTGCTGC	qRT-PCR
	qRT-PCR-00752-R	TGTGTCAACCGTCTTGCCCT	qRT-PCR
VDAG_07210	qRT-PCR-07210-F	CGCCGGTATCTTCGTCGTCT	qRT-PCR
	qRT-PCR-07210-R	CTTGGCGACACCCATGTTGG	qRT-PCR
VDAG_05580	qRT-PCR-05580-F	AGAAGACGGCCCAGCTCATC	qRT-PCR
	qRT-PCR-05580-R	GCCGTGAATACCCTCGCTCT	qRT-PCR
VDAG_09703	qRT-PCR-09703-F	GGACCCAAGATGCGCTTTGG	qRT-PCR
	qRT-PCR-09703-R	GGTCCATGCTCCGGGCTATT	qRT-PCR
VDAG_00808	qRT-PCR-00808-F	CGAGCGACTTTCTCCGAGGT	qRT-PCR

	qRT-PCR-00808-R	TGTATGCGGTGCTCGGGTAG	qRT-PCR
VDAG_05978	qRT-PCR-05978-F	CGATACGCAGCCCACTCTCA	qRT-PCR
	qRT-PCR-05978-R	CGTGGATGGCGTAGGTGGAT	qRT-PCR
VDAG_06165	qRT-PCR-06165-F	GGCACCCACAACTACCAGGT	qRT-PCR
	qRT-PCR-06165-R	TAGCACCGTTCCAGCCTGAG	qRT-PCR
Ve-ITS1	*Ve-ITS1*-F	AAAGTTTTAATGGTTCGCTAAGA	qRT-PCR
ST-VE1	*ST-VE1*-R	CTTGGTCATTTAGAGGAAGTAA	qRT-PCR
GhUBQ7	*UBQ7*-F	GAAGGCATTCCACCTGACCAAC	qRT-PCR
	UBQ7-R	CTTGACCTTCTTCTTCTTGTGCTTG	qRT-PCR

Underlined segments indicate vector homologous arms used for subsequent cloning recombination

Supplementary Figure S1.

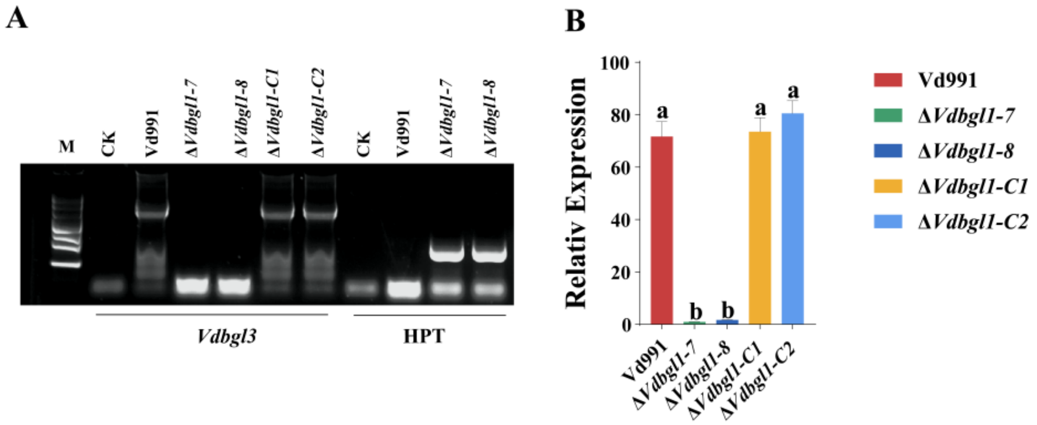

Supplementary Figure S1. Knockout and complementation of the *Vdbgl1* gene in *V. dahliae*

Figure S1. (A) PCR detection of *Vdbgll1* and HPT marker. (B) qRT-PCR analysis of *Vdbgll1* expression.M: 3000 bp DNA marker.

Supplementary Figure S2.

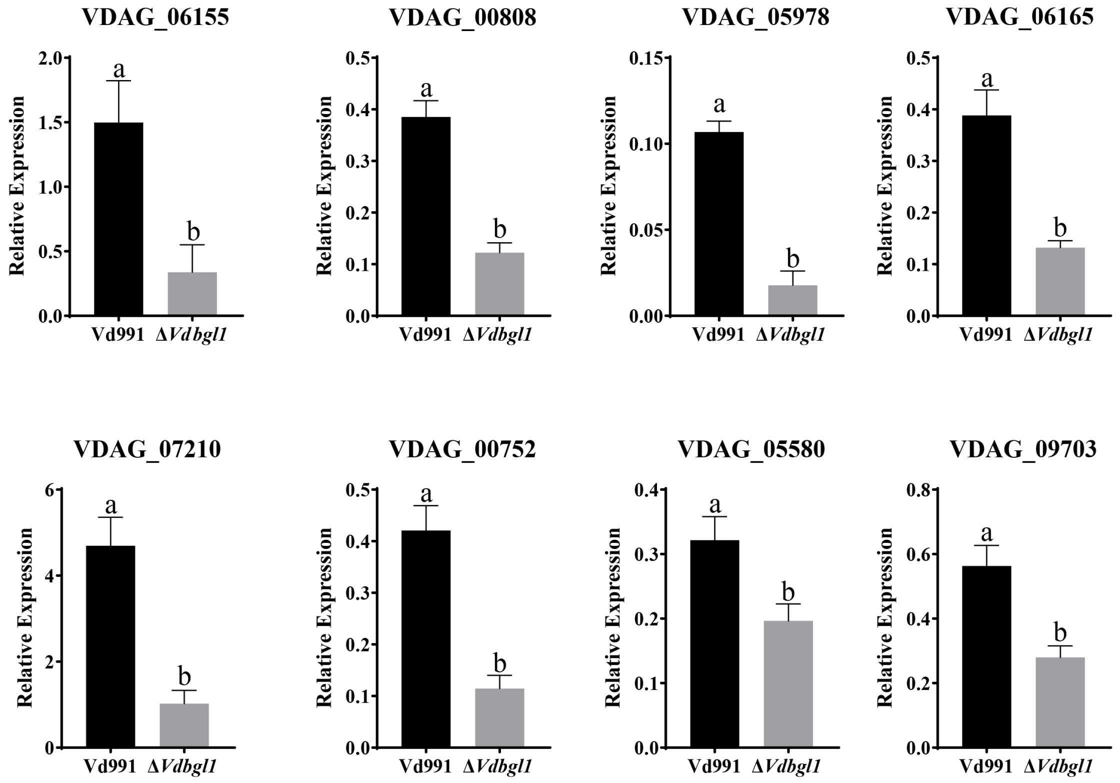

Supplementary Figure S2. qRT-PCR validation of down-regulated differentially expressed genes from transcriptome data. The relative expression levels of 8 down-regulated differentially expressed genes (VDAG_06155, VDAG_00808, VDAG_05978, VDAG_06165, VDAG_07210, VDAG_00752, VDAG_05580 and VDAG_09703) were detected. All experiments were independently repeated at least three times, and data are presented as mean ± standard deviation (Mean ± SD). Statistical analysis was performed using IBM SPSS Statistics 26.0. Significance was assessed via one-way ANOVA followed by Duncan's multiple range test. Different lowercase letters above the bars indicate significant differences between treatments at the $p < 0.05$ level.
